# DNA methylation profiles in chronic lymphocytic leukemia patients treated with chemoimmunotherapy

**DOI:** 10.1186/s13148-019-0783-1

**Published:** 2019-12-02

**Authors:** Maria Tsagiopoulou, Nikos Papakonstantinou, Theodoros Moysiadis, Larry Mansouri, Viktor Ljungström, Martí Duran-Ferrer, Andigoni Malousi, Ana C. Queirós, Karla Plevova, Sujata Bhoi, Panagoula Kollia, David Oscier, Achilles Anagnostopoulos, Livio Trentin, Matthias Ritgen, Sarka Pospisilova, Niki Stavroyianni, Paolo Ghia, Jose I. Martin-Subero, Christiane Pott, Richard Rosenquist, Kostas Stamatopoulos

**Affiliations:** 1 Institute of Applied Biosciences, Center for Research and Technology Hellas, 6th km Charilaou-Thermi Rd, 57001 Thermi, Thessaloniki, GR Greece; 2 Department of Biology, National and Kapodistrian University of Athens, Athens, Greece; 3 Department of Molecular Medicine and Surgery, Karolinska Institutet, Stockholm, Sweden; 4 Department of Immunology, Genetics and Pathology, Science for Life Laboratory, Uppsala University, Uppsala, Sweden; 5 Institut d’Investigacions Biomèdiques August Pi i Sunyer (IDIBAPS), Departamento de Fundamentos Clínicos, Universitat de Barcelona, Barcelona, Spain; 6 Laboratory of Biological Chemistry, Medical School, Aristotle University of Thessaloniki, Thessaloniki, Greece; 7 Department of Internal Medicine–Hematology and Oncology, University Hospital Brno and Medical Faculty of Masaryk University, Brno, Czech Republic; 8 Central European Institute of Technology, Masaryk University, Brno, Czech Republic; 9 Department of Haematology, Royal Bournemouth Hospital, Bournemouth, UK; 10 Hematology Department and HCT Unit, G. Papanicolaou Hospital, Thessaloniki, Greece; 11 Department of Medicine, Hematology and Clinical Immunology Branch, Padua University School of Medicine, Padua, Italy; 12 Second Medical Department, University Hospital Schleswig-Holstein, Campus Kiel, Kiel, Germany; 13 Division of Experimental Oncology and Department of Onco-Hematology, IRCCS San Raffaele Scientific Institute and Università Vita-Salute San Raffaele, Milan, Italy; 14 Institució Catalana de Recerca i Estudis Avançats (ICREA), Barcelona, Spain

**Keywords:** DNA methylation, Chemoimmunotherapy, CLL, Microarray analysis, Relapse

## Abstract

**Background:**

In order to gain insight into the contribution of DNA methylation to disease progression of chronic lymphocytic leukemia (CLL), using 450K Illumina arrays, we determined the DNA methylation profiles in paired pre-treatment/relapse samples from 34 CLL patients treated with chemoimmunotherapy, mostly (*n* = 31) with the fludarabine-cyclophosphamide-rituximab (FCR) regimen.

**Results:**

The extent of identified changes in CLL cells versus memory B cells from healthy donors was termed “epigenetic burden” (EB) whereas the number of changes between the pre-treatment versus the relapse sample was termed “relapse changes” (RC). Significant (*p* < 0.05) associations were identified between (i) high EB and short time-to-first-treatment (TTFT); and, (ii) few RCs and short time-to-relapse. Both the EB and the RC clustered in specific genomic regions and chromatin states, including regulatory regions containing binding sites of transcription factors implicated in B cell and CLL biology.

**Conclusions:**

Overall, we show that DNA methylation in CLL follows different dynamics in response to chemoimmunotherapy. These epigenetic alterations were linked with specific clinical and biological features.

## Background

Mounting evidence highlights a significant contribution of DNA methylation in the onset and evolution of CLL [1]. To large extent, the CLL methylome shares common features with the normal B cell differentiation program [2, 3]. As a whole, CLL cells resemble memory B cells, exhibiting great similarity with the DNA methylation programming for high-maturity memory B cells [3]. Against the initial view that the DNA methylome remains relatively stable, more recent evidence indicates that DNA methylation may evolve overtime, also with respect to treatment [2, 4, 5]. Indeed, patients with mutated IGHV genes (M-CLL) who experience stable disease also display stable DNA methylation patterns overtime, along with limited genetic changes [2]. In contrast, patients with unmutated IGHV genes (U-CLL) appear to show a more wide variation in DNA methylation changes over time [2, 4] along with the appearance of subclones with different genetic aberrations [4]. Despite this evidence, however, the epigenomic contribution to CLL progression remains to be conclusively defined.

The fludarabine-cyclophosphamide-rituximab (FCR) regimen is a standard treatment option for medically fit CLL patients, excepting those who carry aberrations of the *TP53* gene [mutation and/or del(17p)] [6–9]. In fact, along with the somatic hypermutation status of the clonotypic IGHV genes, they are the main predictors of response to FCR treatment in CLL [7–9]. Although FCR is effective with overall response rates in the range of 90%, most patients will eventually relapse with those relapsing within 2 years after FCR experiencing a particularly aggressive disease (“ultra high risk”) [10, 11]. These observations point to a characteristic resistance of the malignant cells that is still not fully characterized from the epigenetic perspective and cannot be reliably predicted, at least for cases not falling in any of the risk categories defined by genetic or immunogenetic biomarkers.

In the present study, we analyzed the DNA methylomes in longitudinal pre-treatment and post-relapse samples of 34 CLL patients treated with chemoimmunotherapy in order to address if changes occur overtime in relation to therapy. We report that DNA methylation profiles are modulated during CLL evolution, particularly in response to treatment, involving several transcription factors and displaying association with particular genetic aberrations. Moreover, we evaluated the DNA methylation alterations occuring during neoplastic expansion, the so-called “epigenetic burden,” comparing the pre-treatment profiles per case with those of memory B cells from healthy donors and highlight that the EB clusters in specific genomic regions and chromatin states, including regulatory regions contaning binding sites of transcription factors implicated in B cell and CLL biology.

## Results

### Genome-wide profiling reveals significant heterogeneity of DNA methylation evolution in CLL

We assessed 68 paired samples from 34 CLL cases who received chemoimmunotherapy as first-line treatment: Of these, 31 were treated with the FCR regimen whereas one each of the remaining 3 cases received FC (fludarabine-cyclophosphamide), FCMR (fludarabine-cyclophosphamide-rituximab+mitoxantrone), or BR (bendamustine-rituximab) (Additional file 1: Table S1). Data from 20/34 pre-treatment CLL samples have been previously reported by our group [12]. The time-span between the two examined states (pre-treatment/post-relapse) ranged from 0.75 to 10.9 years with a median of 2.5 years (Table 1). The vast majority of cases concerned U-CLL (29/34, 85.2%), which are associated with adverse prognosis and significantly higher incidence of progressive disease requiring treatment. First, we performed unsupervised hierarchical clustering based on the methylation levels of 451,756 CpG sites across all samples: This analysis did not discriminate the pre-treatment samples from the post-relapse ones (Additional file 2: Figure S1). Next, we performed differential methylation analysis (DMA) at cohort level (|db| ≥ 0.3, *p* < 0.05 or |db| ≥ 0.3, FDR < 0.05), comparing all the pre-treatment versus all the post-relapse samples, again not identifying differentially methylated CpG (DMCpG) sites.

**Table 1:** Clinical data and results of the intra-individual DNA methylation analysis

Patient ID	U-CLL	IGHV gene	Age at diagnosis	First-line treatment	Treatment response	TTFT	TTR	Cytogenetic alterations*	Pearson *R* (MBC vs pre-treatment)	Epigenetic burden	Pearson *R* (pre-treatment vs relapse)	Relapse changes
P1	NO	IGHV3-11	48	FCR	PR	1.6	4.7	None	0.93	32,380	0.99	117
P2	ND	IGHV1-f	61	FCR	CR	1.1	2.6	del(13q)	0.92	33,441	0.96	20,440
P3	YES	IGHV3-33	55	FCR	CR	0.1	2.3	None	0.93	34,285	0.82	81,383
P4	YES	IGHV1-69	60	FCR	CR	4.3	5.5	Trisomy 12	0.93	35,098	0.98	8052
P5	YES	IGVH1-69	60	FCR	PR	1.4	1.4	del(13q)	0.92	36,696	0.98	3430
P6	YES	IGHV3-33	52	FCR	CR	4.1	6.4	del(13q)	0.92	37,146	0.99	3441
P7	YES	IGHV4-39	62	FCR	CR	2.2	3.6	del(11q), del(13q)	0.92	37,465	0.89	58,299
P8	YES	IGHV1-69	46	FCR	PR	0.3	1.7	del(11q)	0.92	38,023	0.99	1514
P9	YES	IGHV4-31	64	FCR	CR	4.1	3.8	None	0.91	38,652	0.92	40,706
P10	YES	IGHV1-69	61	FCR	CR	1.2	2.2	del(11q)	0.91	39,164	0.97	8199
P11	YES	ND	56	BR	CR	2	3.9	del(13q)	0.92	39,658	1.00	45
P12	YES	IGHV5-a	73	FCR	CR	0.2	3.8	ND	0.92	40,403	0.87	60,970
P13	YES	IGHV3-20	49	FCΜR	PR	2.5	4.1	Trisomy 12	0.91	40,669	0.99	511
P14	YES	ND	29	FCR	PR	1.5	4.8	del(11q), del(13q)	0.91	40,755	0.99	1263
P15	YES	IGHV1-69	66	FCR	CR	3.4	0.9	del(11q), del(13q)	0.91	41,333	0.99	261
P16	YES	IGHV4-34	44	FCR	CR	2.7	1.4	del(11q)	0.91	41,399	0.99	392
P17	YES	ND	36	FCR	CR	2.2	5.0	None	0.90	41,875	0.98	3150
P18	YES	IGHV3-64	26	FCR	CR	0.9	1.3	del(17p)	0.91	42,313	0.99	949
P19	NO	IGHV1-69	48	FCR	CR	6.1	1.8	del(13q)	0.92	42,384	1.00	45
P20	YES	IGHV1-69	61	FCR	ND	0	1.1	del(6q)	0.91	42,543	0.99	457
P21	YES	IGHV1-24	59	FCR	CR	0	1.2	None	0.91	42,615	0.99	907
P22	YES	IGHV3-30	56	FCR	CR	0	0.8	del(11q)	0.91	42,721	0.99	287
P23	YES	IGHV1-69	54	FCR	CR	1.5	0.9	del(11q), del(13q)	0.90	44,283	0.99	90
P24	NO	IGHV1-69	ND	FCR	CR	4.7	1.2	del(11q , del(13q)	0.91	45,253	1.00	45
P25	YES	IGHV3-48	65	FCR	CR	2	8.0	del(13q)	0.91	45,372	0.99	905
P26	YES	IGHV1-69	64	FCR	CR	0.1	2.2	del(11q)	0.89	45,383	0.98	4689
P27	ND	IGHV3-53	43	FCR	CR	0.1	10.9	Trisomy 12	0.89	45,999	0.96	10,449
P28	YES	IGVH3B	61	FCR	CR	0.1	1.4	del(17p)	0.90	46,314	0.89	51,925
P29	YES	IGHV4-34	53	FCR	CR	0	7.9	None	0.90	50,072	0.98	8546
P30	YES	ND	55	FC	CR	2	3.0	del(11q)	0.89	50,443	0.98	2740
P31	YES	IGVH3B	50	FCR	CR	0.1	1.5	del(13q)	0.89	52,336	1.00	57
P32	YES	IGHV3-48	70	FCR	CR	0.2	5.1	del(11q)	0.89	53,793	0.98	4504
P33	YES	IGVH3A-3B	59	FCR	CR	0	2.1	del(6q)	0.86	59,041	0.97	6584
P34	YES	IGHV1-3	38	FCR	PR	1	3.0	del(13q)	0.89	60,526	0.98	2985

*TTFT* time to first treatment (years) *TTR* time to relapse (years)*, ND* not determined. *Detected by karyotype and/or FISH (pre-treatment state)

Next, we proceeded to methylation analysis at the individual case level. In addition to the DNA methylation changes observed after relapse, we also investigated the alterations which had occurred in comparison to normal B cells. We used as reference available data from two samples of peripheral blood memory B cells (MBC) from healthy donors recently reported by our group [12]. We found high correlation between the biological replicates (Pearson *R* = 0.99) of MBCs; thus, we merged and used them as a single reference sample.

**Fig. 1:**
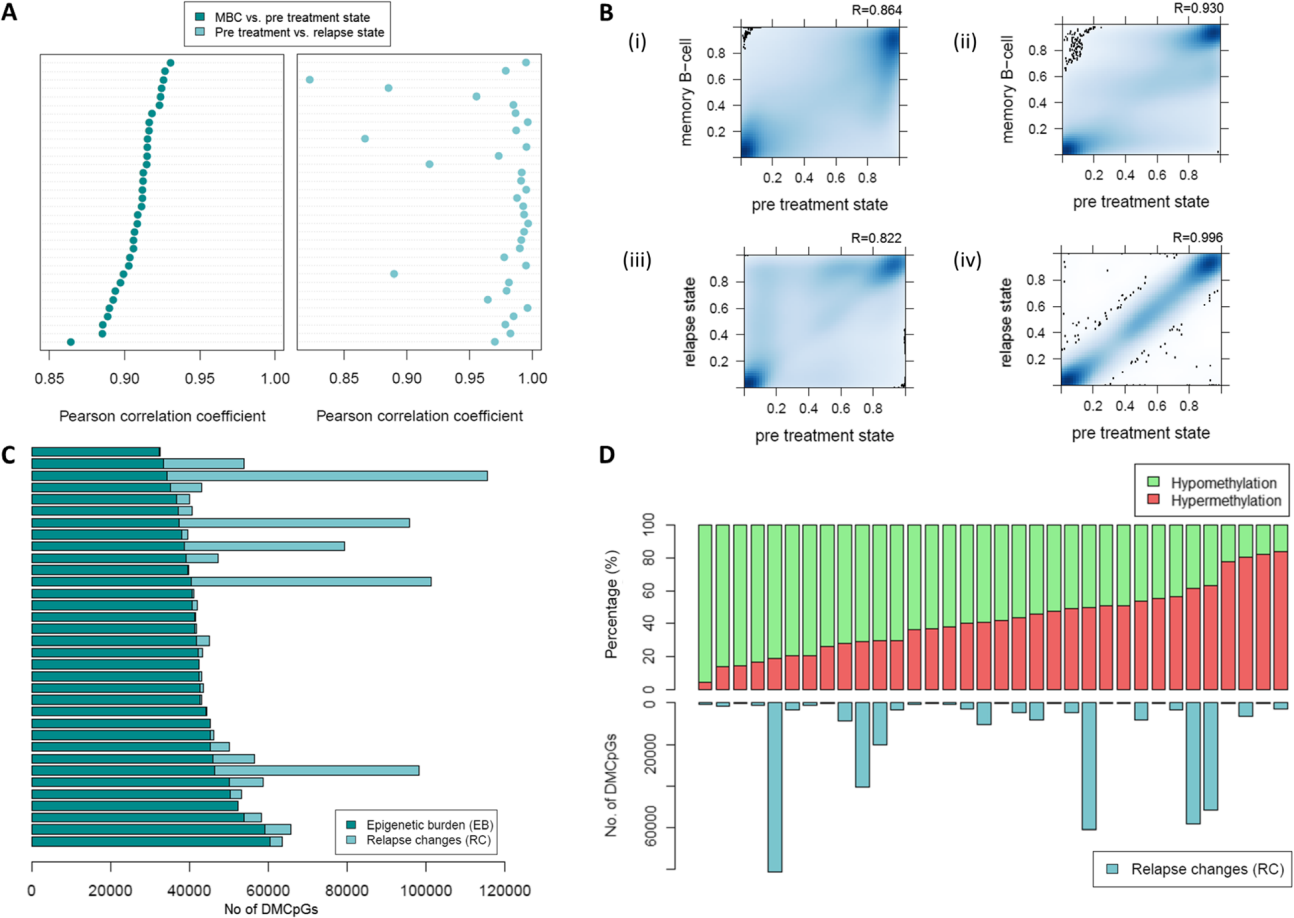
Intra-individual DNA methylation analysis reveals significant variability of epigenetic burden and relapse changes between CLL cases. **a** Dot plots showing the Pearson coefficient values (*x*-axis) representing the correlation of the MBC with the pre-treatment *b* values (left part, dark blue) and the correlation of the pre-treatment with the relapse *b* values (right part, light blue) of each CLL case (*n* = 34). **b** Density heatmaps displaying the frequencies of the *b* values for four selected CLL cases which showed the minimum and maximum Pearson *R* value after comparison of the *b* values of MBC (*y*-axis) and the pre-treatment state (*x* axis) which are depicted in (i) and (ii), and the *b* values of the relapse (*y*-axis) and pre-treatment state, which are depicted in (iii) and (iv). **c** Barplot, with each bar representing a different CLL case and showing the total number of DMCpGs related to the epigenetic burden (dark blue) plus the total number of DMCpGs related to relapse (light blue). **d** Barplots showing the percentage of hypermethylated (red color) and hypomethylated (green color) CpG sites at the relapse compared to the pre-treatment state, herein defined as relapse changes (RC), for all cases analyzed (upper part). The barplots in the lower part show the total number of the RCs for each case. Bars represent individual CLL cases

First, we compared the MBC versus the pre-treatment sample for each CLL case and noticed a variability in the Pearson *R* values ranging from 0.864 to 0.930: The number of DMCpGs (|db| ≥ 0.3), referred to as epigenetic burden (EB), ranged from 32,380 to 60,526 (Fig. 1a–c) (Table 1, Additional file 1: Table S1). All 34 CLL cases showed massive hypomethylation compared to MBC, in keeping with previous reports [13, 14] (Additional file 2: Figure S2).

Next, we compared the pre-treatment sample versus the relapse sample for each case and observed that the number of DMCpGs (|db| ≥ 0.3), referred to as relapse changes (RC), showed significant variability among patients ranging from 45 to 81,383 (Pearson *R* 0.822–0.996) (Fig. 1a–c) (Table 1, Additional file 1: Table S1). Twenty-three of the 34 (68%) samples after relapse showed a higher number of hypomethylated than hypermethylated CpGs compared to the pre-treatment state (Fig. 1d).

Interestingly, we observed an overlap between the EB and RC, with the number of overlapping CpGs ranging from 13 to 22,853. Next, in order to examine the persistence of specific CpG sites affected at relapse, we calculated the number of overlapping CpGs/RC (Additional file 2: Figure S3). We found that most cases showed > 20% overlap between RC and EB at relapse, implying that DNA methylation changes, at least in part, occur in specific regions.

### DNA methylation changes in CLL cluster in specific genomic regions, chromatin states, and transcription factor binding sites

**Fig. 2:**
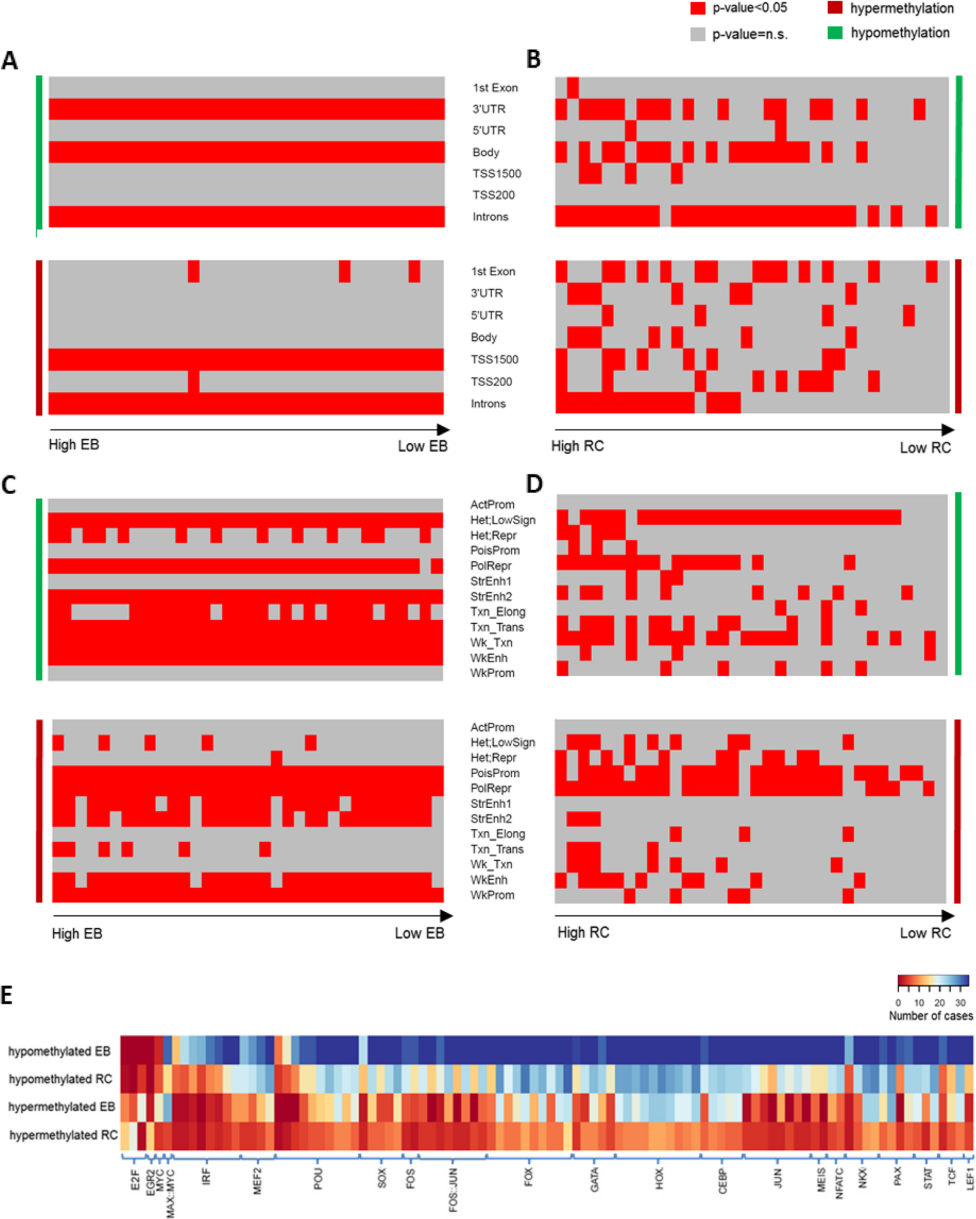
Enrichment analysis of the epigenetic burden and relapse changes regarding genomic locations, chromatin states, and transcription factor binding sites. Genomic location enrichment analysis of **a** the epigenetic burden hypomethylated (green) and hypermethylated (red) CpGs for each CLL case and **b** the relapse hypomethylated (green) and hypermethylated (red) CpGs for each CLL case. Chromatin states enrichment analysis of **c** the epigenetic burden hypomethylated (green) and hypermethylated (red) CpGs for each CLL case and **d** the relapse hypomethylated and hypermethylated (red) CpGs for each CLL case. Each column represents a CLL case, with the cases sorted on *x*-axis based on the number of DMCpGs per case, from maximum to minimum. Each row represents a genomic element. The red color on heatmap displays the significant enrichment (*p* < 0.05) in each case for the respective genomic element (ActProm, active promoter; Hete LowSign, heterochromatin low signal; Het Repr, heterochromatin-repressed; PolRepr, polycomb repression; PoisProm, poised promoter; StrEnh1, strong enhancer 1; StrEnh2, strong enhancer 2; Txn_Elong, transcription elongation; Txn_Trans, transcription transition, Wk_Txn, weak transcription; WkEnh, weak enhancer; WkProm, weak promoter. **e** TFBS analysis of the hypo- and hypermethylated epigenetic burdens and relapse changes revealed significant enrichment for several TFs families (*x*-axis). The density of heatmap represents the number of patients which showed statistical significant enrichment per TFBS (FDR < 0.05)

We characterized the DMCpGs detected in each CLL case regarding both the EB and the RC based on: (i) their genomic location, (ii) the respective chromatin state of MBC, and (iii) the transcription factor binding sites (TFBS) and performed enrichment analyses in order to gain insight into the biological function of the observed changes. Regarding genomic location and the chromatin state, we observed that the EB-hypomethylated CpGs were enriched in introns, gene bodies, and 3’ UTRs placed mainly in heterochromatin and strong and weak enhancers, while the EB hypermethylated CpGs were enriched in introns and TSS upstream regions (TSS1500) mostly located within polycomb repressed regions, poised and weak promoters, and strong enhancers in all 34 cases (Fig. 2a, c). The RC DMCpGs showed similarities as well as differences from the EB DMCpGs. More specifically, the RC-hypomethylated CpGs showed enrichment in introns, gene bodies, and 3’ UTRs as observed also in EB, but they were placed mainly in heterochromatin with only few cases placed in enhancers. Regarding the RC-hypermethylated CpGs, no consistent pattern was shared between cases. They preferentially clustered to the first exons, introns, and TSS upstream regions, which were located to poised promoters and polycomb-repressed regions, while enhancers were almost absent (Fig. 2b, d).

TFBS analysis revealed that both EB- and RC-hypomethylated CpGs were enriched (FDR < 0.05) for binding sites of a large series of TFs (Fig. 2e, Additional file 3: Table S2, and Additional file 4: Table S4). More specifically, in all cases, the EB-hypomethylated regions showed enrichment for several TFs relevant to B cell/CLL biology, including members of *AP-1*, *GATA*, *IRF*, *POU*, *NFAT*, *STAT*, and *TCF* families, as well as most members of HOX and FOX development-related TFs. This pattern was also observed on RC-hypomethylated regions, albeit in a lower number of analyzed cases (Fig. 2e, Additional file 3: Tables S2 and Additional file 4: Table S4). The EB- and RC-hypermethylated regions showed enrichment for TFBS in fewer CLL cases compared to the EB- and RC-hypomethylated regions, respectively (Fig. 2e, Additional file 5: Table S3, Additional file 6: Table S5 and Additional file 7: Table S6). Both hypo- and hypermethylated regions were enriched for TFBS of HOX and FOX families in contrast to TFBS such as *FOS*, *JUN*, *IRF*, and *CEBP*, which were specific for hypomethylated regions (Fig. 2e, Additional file 7: Table S6). Interestingly, the RC-hypermethylated regions were enriched for EGR2 and E2F4 binding sites in a large number of CLL cases (*n* = 14 and *n* = 18, respectively); however, most analyzed cases did not show similar enrichment for RC-hypomethylated or EB- (both hyper- and hypomethylated) regions (Additional file 7: Table S6).

Finally, KEGG pathway enrichment analysis based on the DM genes, showed that, in almost all cases, EB-hypomethylated and hypermethylated-regions were enriched for pathways significant for CLL biology, e.g., *ErbB*, *Phospholipase D*, *Ras*, *HIF*, *MAPK*, *Wnt*, *T*, and *B cell receptor*, and *Notch* signaling pathways (Additional file 8: Table S7 and Additional file 9: Table S8). In contrast, a similar analysis for RC revealed enrichment in only a fraction of analyzed cases, especially those with a high number of changes, mainly on the hypermethylated gene sets (Chi-squared test, *p* < 0.05), e.g., pathways in cancer, calcium signaling pathway, and *Rap1* signaling pathway (Additional file 9: Table S8).

### DNA methylation changes overtime associate with specific biological characteristics and clinical outcome

Based on the results of the intra-individual methylation analysis, where each CLL case showed a different number of affected CpG sites compared to both the MBC and the relapse state, we explored potential correlations of the EB and RC with clinical and molecular characteristics.

First, considering the fact that the DNA methylation profiles are affected by aging [15], we investigated the correlation between the age of patients with EB and RC, however, not finding any significant correlation (Spearman rho between RC and age = − 0.28, *p* = 0.11 | Spearman rho between EB and age = 0.118 *p* = 0.50).

**Fig. 3:**
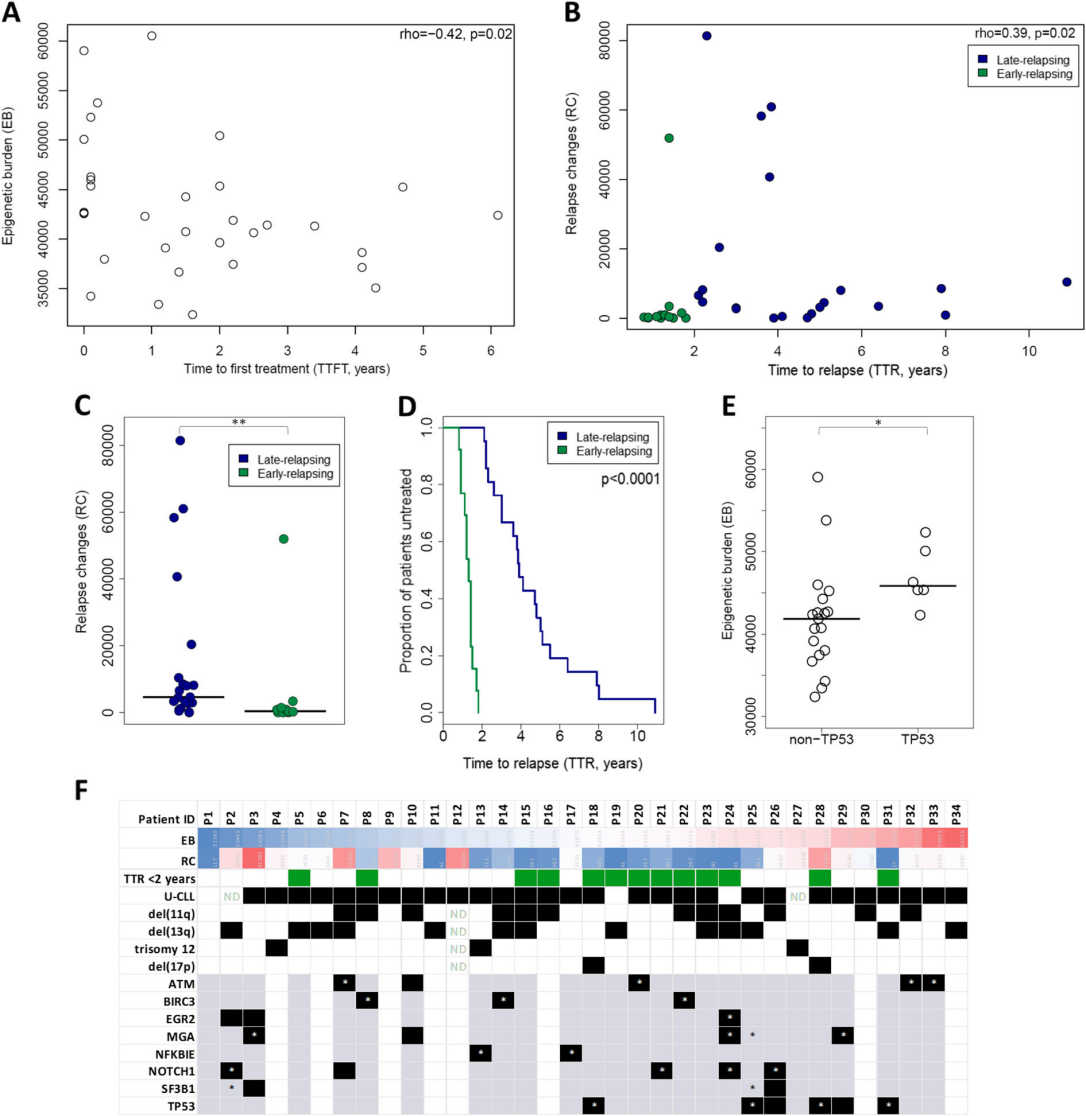
Associations of the epigenetic burden and the relapse changes with clinicobiological characteristics. **a** Scatter plot showing the epigenetic burden (EB) (*y*-axis) and the time to first treatment (TTFT) (*x*-axis) and their correlation coefficients (Spearman rho = 0.42, *p* = 0.02) for all 34 CLL cases **b** Scatter plots showing both the relapse changes (RC) (*y*-axis) and the time to relapse (TTR) (*x*-axis) and their correlation coefficients (rho = 0.39, *p* = 0.02) for all 34 CLL cases. The early-relapsing cases are displayed with green and the late-relapsing cases with blue. **c** Dot plot with median showing the RC of the early-relapsing and late-relapsing groups which show significant differences (*p* < 0.001). **d** Kaplan-Meier curves for time to relapse (TTR), with the early-relapsing cases relapse significant earlier than the late-relapsing (Log-test, *p* < 0.001). **e** Dot plot with median showing the EB of CLL cases with TP53 and non-TP53 aberrations which display significant differences (*p* < 0.05). **f** Columns represent patients (*n* = 34) and rows genes or cytogenetic abnormalities. The color scale represents the number of DMCpGs from blue (low) to red (high). Regarding cytogenetic abnormalities, black boxes state the presence of the aberration and white boxes the absence. Regarding gene mutations, gray boxes state the existence of whole-exome sequencing (WES) data available for the respective genes and the absence of mutation; gray boxes with asterisk (*) state the presence of mutation detected only at the pre-treatment state, black boxes the presence of mutation at relapse, and black boxes with asterisk represent the presence of mutation detected both at pre-treatment and at relapse state. White boxes state no availability of WES data. ND, not available data; **p < 0.01, *p < 0.05

Next, we noticed a significant inverse correlation between the EB and the time to first treatment (TTFT) (Spearman rho = − 0.42, *p* = 0.02) (Fig. 3a); no such correlation was identified with the time-to-relapse (TTR) (Spearman rho = − 0.112, *p* = 0.52). Moreover, we observed a significant positive correlation between the RC and the TTR (Spearman rho = 0.39, *p* = 0.02) (Fig. 3b). Relevant to mention, 13 of 34 cases could be characterized as early-relapsing since they relapsed within 2 years of treatment; notably, these cases displayed low or no evolution of DNA methylation. Put differently, we found very few RC in the early-relapsing cases compared to the rest which relapsed after 2 years of treatment, hereafter referred to as the late-relapsing group (median of DMCpGs 39 vs 4689, *p* = 0.002) (Fig. 3c), which, not unexpectedly, showed significantly longer TTR (median TTRs 1.3 vs 3.9 years, *p* = 4.32e-11).

Next, we examined potential associations of epigenetic evolution regarding the EB and RC with particular biological characteristics of the malignant clones. Considering that the vast majority of cases (29/34, 85.2%) concerned U-CLL, we did not explore associations with immunogenetic features [i.e., the IGHV gene somatic hypermutation status] and, instead, decided to focus on genomic aberrations. To this end, we used available cytogenetic data (FISH and/or karyotype) regarding the Dohner model abnormalities [i.e., del(11q), del(13q), del(17q), and trisomy 12] for all 34 cases. Moreover, we used existing whole-exome sequencing (WES) data for 26/34 cases reported in a recent publication from our group [16] and examined potential associations with mutations within the *ATM*, *BIRC3*, *EGR2*, *MGA*, *NFKBIE*, *NOTCH1*, *SF3B1*, and *TP53* genes. Additionally, for 16/26 cases, we had available data for clonal evolution (all mutations were documented at both pretreatment and relapse and assigned to separate clusters using the SciClone25 clustering tool). Interestingly, cases carrying *TP53* aberrations showed significantly higher EB versus *TP53*-wildtype CLL cases (median-EB = 45,848.5 vs 42,384, respectively, *p* = 0.0176) (Fig. 3d). Additionally, most cases with available data (14/16), previously published [16], showed relapse-specific subclones/clusters which expanded significantly to become the dominant clone at relapse; the remaining 2 cases showing a more stable intraclonal composition over time accompanied by no epigenetic evolution after relapse (RC = 117 and 1263). Relevant to mention, the cases analyzed in our study were treated with FCR at a time when signaling inhibitors, currently considered as the standard of care for *TP53* aberrant cases, were not available; hence, a proportion of cases who received FCR treatment carried such aberrations. All the examined prognostic markers are summarized graphically in Fig. 3e while the analysis regarding their association with the EB and RC is depicted in Additional file 2: Figures S4-S5.

## Discussion

Recent studies have demonstrated extensive genetic heterogeneity underlying clonal evolution in CLL patients with adverse clinical outcomes [16, 17]. Besides genetic events, however, CLL is also characterized by epigenetic alterations, with DNA methylation profiles being the best studied thus far [2, 14, 18–20]. Interestingly, a recent study demonstrated that CLL cases belonging to the memory B-cell-like epigenetic subgroup [21] showed a favorable response to FCR [22]. However, insight into how the epigenetic mechanisms might be implicated in disease evolution, particularly the response to treatment, is still lagging.

In order to obtain evidence regarding this issue, we analyzed longitudinal samples from a series of 34 CLL patients mostly (*n* = 31) treated with the FCR regimen in order to explore DNA methylation changes overtime and whether these may be associated with the patterns of clinical response. At cohort level, we did not observe recurrent DNA methylation changes, in line with two previous studies [2, 4]. This could be attributed, at least in part, to the large heterogeneity characterizing the DNA methylation profile of CLL cases [23, 24], even those carrying IGHV genes with concordant somatic hypermutation status [12] as in the present series, where the vast majority of cases concerned U-CLL.

Considering the above, we focused on each individual patient separately and examined both the epigenetic burder (EB), i.e., the methylation changes that tumor cells acquire compared to memory B cells form healthy donors (considering that all CLL cases globally resemble memory B cells) [3] but also the changes between the pre-treatment sample versus the relapse sample, referred to as relapse changes (RC). This intra-individual analysis confirmed once again the pronounced biological heterogeneity of CLL, since great variability was noted between cases regarding both the EB but also the RC. In all cases, CLL cells were characterized by extensive epigenetic reprogramming displaying massive hypomethylation compared to memory B cells, in keeping with previous studies [3, 14, 25]. Relevant to mention, it was recently shown that the number of epigenetic changes that a tumor acquires compared to its cellular origin, i.e., the EB, may be a powerful predictor of clinical aggressiveness in mantle cell lymphoma (MCL) [26] where patients with high EB experienced a worse clinical outcome. Following the same approach, we here report a similar observation also for CLL, since we found that the higher the EB the shorter the TTFT.

Regarding the RCs, we found very high inter-patient variability; some cases had stable epigenetic profiles while others showed significant epigenetic evolution after relapse, with a mixed pattern of hypomethylated or hypermethylated events at relapse. Notably, the number of RCs was positively correlated with the time to relapse. Especially, those cases relapsing within 2 years of treatment (“early-relapsing”) displayed low or no evolution of DNA methylation. Our observation appears to contradict a recent publication [4], which reported that epigenetic evolution after treatment is linked with short time to post-therapy events (treatment and death). These discrepant results might be attributed to differences in the composition and size of the respective study cohorts (21 vs 34 cases in our series) as well as the administered treatments (purine analogs and/or alkylating agents vs FCR in our series), but also differences in the applied data analysis strategies.

In an attempt to identify a unifying line of events, we sought for connections regarding the methylation changes occurring overtime and also explored associations with genomic alterations and other clinicobiological features. Our results suggest that most CLL cases carrying *TP53* aberrations display higher EB compared to the rest and confirm the association of higher EB with shorter TTFT. This finding links adverse-prognostic genomic aberrations with a higher propensity to evolve epigenetically, perhaps as a consequence of high rates of proliferation [27–29]. Such epigenetic evolution might potentially facilitate subclonal expansion as also suggested by a previous study which showed that a high level of alterations in DNA methylation was accompanied with a greater probability to develop new subclones [23]. Admittedly, one could also argue for the opposite, namely that the genomic evolution should fuel the epigenomic evolution, especially considering the well-known complex interplay between genetics and epigenetics whereby the genomic instability could influence the epigenetic evolution and vice versa [30, 31]. Relevant to mention in this respect, p53 gain of function mutants have been found to bind to and upregulate chromatin regulatory genes that may influence the DNA methylation patterns [32]. Turning to RC, we observed significant correlation with TTR but also great heterogeneity. Most early-relapsing cases showed low or no evolution of the respective DNA methylation profiles suggesting that these cases did not have sufficient time to accumulate DNA methylation changes and/or were more progressive. In such cases, treatment with FCR does not appear to impact significantly on the clonal behavior, leading to the re-emergence of a clone that is not fundamentally different from the pre-treatment clone, at least at the level of resolution of our study.

From a qualitative point of view, the majority of methylation changes at relapse appear to follow a pattern where hypomethylation events cluster mainly in gene bodies and heterochromatic regions while hypermethylation events cluster in promoters and polycomb-related regions. This pattern is also observed during B cell differentiation (e.g., long-lived memory/plasma cells) as well as in other tumors and may represent a passive result of, e.g., proliferation history and cellular longevity [14, 25, 33]. Relevant to mention, in a previous study from our group, we found that the histone methyltransferase *EZH2*, the catalytic subunit of the polycomb repressive complex 2 (PRC2), is upregulated during the disease course in CLL, especially at relapse [34]. In another recent study, Smith and colleagues [5] identified modest recurrent DNA methylation changes during CLL progression in CpG sites enriched for regions near targets of the PRC2 complex.

Substantial evidence supports an interplay between transcription factors and DNA methylation both during normal B cell development but also during the course of CLL [3, 14, 25]. In the present study, we observed that genomic regions which became hypomethylated prior to treatment initiation but also after relapse were enriched for binding sites of several transcription factor families relevant to B cell/CLL biology, including the *GATA*, *STAT*, *HOX*, and *FOX* transcription factors (TFs). Among others, we also observed *AP-1*, *POU*, and *IRF* which were descripted to target hypomethylated regions during normal B cell maturation [3] and also the NFAT family which has been associated with hypomethylated regions in CLL [3, 35]. Interestingly, de novo active chromatin regions in CLL compared to normal B cells were very recently reported to be enriched in FOX TF family binding sites as well as the *NFAT* and *TCF/LEF* TFs [36]. On the other hand, *EGR2* and *E2F4* were found to be specifically associated with regions hypermethylated at relapse, implying a direct connection with the relapse mechanism. Of note, *EGR2* mutations have been detected in clinically aggressive CLL subgroups [37] while *E2F4*, a key regulator of the cell cycle, has been reported deregulated in both Burkitt lymphoma and diffuse large B cell lymphoma [38].

## Conclusions

In conclusion, this study highlights that DNA methylation profiles are modulated during CLL evolution, particularly in response to chemoimmunotherapy with the FCR regimen. These distinct dynamics of DNA methylation were linked to clinicobiological characteristics, including genomic aberrations, time-to-first-treatment, and response to treatment as assessed by time-to-relapse. These alterations mainly occurred in specific genomic regions, following a pattern similar to that observed in long-lived memory/plasma cells and other tumor types, while they were associated with binding sites of transcription factors implicated in B cell and CLL biology.

## Methods

### Patient samples

Eighty peripheral blood (PB) samples from 40 CLL patients from 7 collaborating institutions in the Czech Republic, Germany, Greece, Italy, Sweden, and the UK were included in the study. All cases were diagnosed with CLL according to the guidelines of the International Workshop Chronic Lymphocytic Leukemia/National Cancer Institute (iwCLL/NCI) [39]. The first sample for each patient was collected before the first treatment and is characterized as the pre-treatment sample while the second soon after clinically documented relapse and is characterized as the post-treatment (i.e., after relapse) sample. Thirty-six of the 40 patients were treated with FCR, 2/40 with FC, and one each of the remaining 2 cases with BR or FCMR. The study was approved by the local Ethics Review Committee of the participating institutions. Demographic, clinical, and biological data for the patient cohort is listed in Additional file 1: Table S1.

### Cell separation

The tumor load of CLL PB samples ranged from 51 to 100%. Purified CLL cell samples (*n* = 46) were prepared with negative selection of CD19^+^ B cells from whole blood using the RosetteSep B-cell enrichment kit (StemCell Technologies, Vancouver, BC, Canada) following the manufacturer’s instructions.

### DNA methylation array analysis

Preparation of DNA samples and processing the DNA methylation signal of the Infinium HumanMethylation 450K BeadChip were performed in *R* using the “minfi” package. We used the subset-quantile within array normalization (SWAN) [40] that corrects for the technical differences between the Infinium I and II assay designs and produces a smoother overall beta value distribution. Moreover, we developed and optimized an analysis pipeline with several filters (i.e., discarding CpGs with low detection *p* values, sex-specific CpGs, CpGs showing individual-specific methylation, and CpGs overlapping with SNPs). Regarding batch effects, we analyzed 10 patient samples in two different batches in order to check the Pearson *R* for each sample between the two batches (Pearson *R* ranging from 0.9932 to 0.9970). Moreover, the in silico purification was performed based on a new approach we previously developed for deconvolution of the DNA methylation signal of mixed subpopulations helping to isolate in silico the DNA methylation levels of the tumor cells [26, 41] (Additional file 2: Figure S6). It is widely acknowledged that CLL cells resemble antigen-experienced B cells, particularly displaying a phenotype more similar to that of memory B cells [42]. Therefore, using memory B cells as a normal counterpart to detect epigenetic changes in CLL patients appears to represent a rational, in fact perhaps the most appropriate, healthy control. With this in mind, we used as reference for normal B cells two samples of peripheral blood memory B cells (MBC) from healthy donors recently reported by our group [12].

### Data availability

The dataset supporting the conclusions of this article is available in the ΕΒΙ repository, https://www.ebi.ac.uk/arrayexpress/experiments/E-MTAB-7575, reference number E-MTAB-7575.

### Genomic and functional annotation of CpGs

The differentially methylated CpG sites (DMCpGs) were characterized based on their genomic locations (e.g., TSS, exon, and intron) and the chromatin states (ChIP-seq for 6 histone marks) from a pool of memory B cells obtained from healthy male donors with age ranging from 56 to 62 years recently published [26] (the above reference for the functional annotation is age-matched with the present study group with a mean age 56.5 years). Moreover, we investigated the overlap between the DMCpGs and the JASPAR database of transcription factor binding sites [43] (Additional file 2: Supplementary Methods). Finally, we performed KEGG-pathway enrichment analysis using the differentially methylated genes.

### Statistical analysis and visualization

Differential methylation analysis (DMA) was performed for the CpG sites of methylation profiles between two conditions. CpG sites were considered as differentially methylated when the following criteria were met, specifically, a minimum absolute difference of 0.3 between mean beta-values (beta difference, db) of the two subgroups and a *p* value criterion when appropriate. We used the Wilcoxon-paired test for paired samples applying FDR for the correction. The Kruskal test was used in a different context for testing differences between independent samples. Data analysis was carried out in the R environment (3.5.1 version). More details can be found in the Additional file 2: Supplementary Methods.

## Supplementary information

**Additional file 1: Table S1.** Clinicobiological data and results of the intra-individual DNA methylation analysis for the patient cohort.

**Additional file 2: **Supplementary Methods**. Figure S1.** A. Hierarchical clustering of all 68 samples, based on the methylation levels of 451756 CpG sites analyzed per case. 30/34 cases showed strong intra-individual similarity. **B.** Principal component analysis showing components 1 and 2 in the pre treatment and post relapse stage based on 451756 CpG sites. **Figure S2.** Barplots showing the percentage of hypermethylated (red color) and hypomethylated (green color) CpG sites revealed after the comparison of the MBC (memory B cells) with the pre-treatment state of each case analyzed. **Figure S3.** Dot plots with median showing the RC in subgroups of CLL cases based on the genomic aberrations. **Figure S4.** Dot plots with median showing the EB in subgroups of CLL cases based on the genomic aberrations. **Figure S5.** Dot plots with median showing the RC in subgroups of CLL cases based on the genomic aberrations. **Figure S6.** Graphical description of the study aim, the study group and the methods used. We performed deconvolution of DNA methylation data, since a part of CLL samples was characterized bythe tumor load <95%. Estimation of the proportion of hematopoietic cell subpopulations in CLL samples and sorted B cells, CD8+ T cells, CD4+ T cells, natural killer cells, monocytes and granulocytes. Sorted cell subpopulations (a right part of the heatmap) are correctly predicted and CLL cases show a gradient from lower to higher proportion of B cells (a left part of the heatmap).

**Additional file 3: Table S2.** TFBS enrichment analysis of the hypomethylated epigenetic burdens revealed common TFs among CLL cases. Each row represents a TFBS and each column represents a CLL case and displays the number of DMCpGs associated with the TFBS. Each patient column is accompanied by one more column representing the p-value after FDR correction regarding the statistical significance according the background. The last column shows the frequency of the enriched cases in each TF. The patients were sorted based on the number of the relapse changes, beggining from the higher to lowest number.

**Additional file 4: Table S4.** TFBS enrichment analysis of the hypomethylated relapse changes revealed common TFs among CLL cases. Each row represents a TFBS and each column represents a CLL case and displays the number of DMCpGs associated with the TFBS. Each patient column is accompanied by one more column representing the p-value after FDR correction regarding the statistical significance according the background. The last column shows the frequency of the enriched cases in each TF. The patients were sorted based on the number of the relapse changes, beggining from the higher to lowest number.

**Additional file 5: Table S3.** TFBS enrichment analysis of the hypermethylated epigenetic burden revealed common TFs among CLL cases. Each row represents a TFBS and each column represents a CLL case and displays the number of DMCpGs associated with the TFBS. Each patient column is accompanied by one more column representing the p-value after FDR correction regarding the statistical significance according the background. The last column shows the frequency of the enriched cases in each TF. The patients were sorted based on the number of the relapse changes, beggining from the higher to lowest number.

**Additional file 6: Table S5.** TFBS enrichment analysis of the hypermethylated relapse changes revealed common TFs among CLL cases. Each row represents a TFBS and each column represents a CLL case and displays the number of DMCpGs associated with the TFBS. Each patient column is accompanied by one more column representing the p-value after FDR correction regarding the statistical significance according the background. The last column shows the frequency of the enriched cases in each TF. The patients were sorted based on the number of the relapse changes, beggining from the higher to lowest number.

**Additional file 7: Table S6.** TFBS enrichment analysis of the hypo- and hypermethylated epigenetic burdens and relapse changes separately, revealed common TFs among CLL cases.

**Additional file 8: Table S7.** KEGG enrichment analysis of the epigenetic burden hypo- and hyper- methylated genes separately, revealed common pathways among CLL cases.

**Additional file 9: Table S8.** KEGG enrichment analysis of the relapse hypo- and hyper- methylated genes separately, revealed common pathways among CLL cases.

## Data Availability

The dataset(s) supporting the conclusions of this article is(are) available in the ΕΒΙ repository, https://www.ebi.ac.uk/arrayexpress/experiments/E-MTAB-7575, reference number E-MTAB-7575.

## Abbreviations

BR: Bendamustine-rituximab
CLL: Chronic lymphocytic leukemia
DMA: Differential methylation analysis
DMCpGs: Differentially methylated CpG sites
EB: Egenetic burden
FC: Fludarabine-cyclophosphamide
FCMR: Fludarabine-cyclophosphamide-rituximab+mitoxantrone
FCR: Fludarabine-cyclophosphamide-rituximab
iwCLL/NCI: International Workshop Chronic Lymphocytic Leukemia/National Cancer Institute
MBC: Memory B cells
M-CLL: Chronic lymphocytic leukemia cases carrying mutated immunoglobulin receptors
PB: Peripheral blood
RC: Relapse changes
TFBS: Transcription factor binding sites
TTFT: Time-to-first-treatment
TTR: Time-to-relapse
U-CLL: Chronic lymphocytic leukemia cases carrying unmutated immunoglobulin receptors
