# Supplementary material for: DNA methylation profiles in chronic lymphocytic leukemia patients treated with chemoimmunotherapy
Source: Clin Epigenetics. 2019 Dec 2;11:177. doi: 10.1186/s13148-019-0783-1 (PMC6889736; doi:10.1186/s13148-019-0783-1)
Supplement: Supplementary file 2 — Additional file 2: Supplementary Methods. Figure S1. A. Hierarchical clustering of all 68 samples, based on the methylation levels of 451756 CpG sites analyzed per case. 30/34 cases showed strong intra-individual similarity. B. Principal component analysis showing components 1 and 2 in the pre treatment and post relapse stage based on 451756 CpG sites. Figure S2. Barplots showing the percentage of hypermethylated (red color) and hypomethylated (green color) CpG sites revealed after the comparison of the MBC (memory B cells) with the pre-treatment state of each case analyzed. Figure S3. Dot plots with median showing the RC in subgroups of CLL cases based on the genomic aberrations. Figure S4. Dot plots with median showing the EB in subgroups of CLL cases based on the genomic aberrations. Figure S5. Dot plots with median showing the RC in subgroups of CLL cases based on the genomic aberrations. Figure S6. Graphical description of the study aim, the study group and the methods used. We performed deconvolution of DNA methylation data, since a part of CLL samples was characterized bythe tumor load <95%. Estimation of the proportion of hematopoietic cell subpopulations in CLL samples and sorted B cells, CD8+ T cells, CD4+ T cells, natural killer cells, monocytes and granulocytes. Sorted cell subpopulations (a right part of the heatmap) are correctly predicted and CLL cases show a gradient from lower to higher proportion of B cells (a left part of the heatmap). [file 13148_2019_783_MOESM2_ESM.doc]

**Supplementary Material**

**Supplementary Methods**

**Genomic location and chromatin states enrichment analysis**

The annotation file from Illumina was used to separate the differential methylated CpG sites (DMCpGs) based on their genomic locations. ChIP-seq data of 6 histone marks (H3K4me1, H3K4me3, H3K27ac, H3K36me3, H3K27me3, H3K9me3) from memory B cells of healthy donors, recently published , were used for the annotation of the DMCpGs to the respective chromatin states. The following regions were considered, adapted from a previously published segmentation : Active promoter (H3K4me3+, H3K27ac+), Weak Promoter (H3K4me1+, H3K4me3+), Poised Promoter (H3K4me1+ , H3K4me3+, H3K27me3+), Strong Enhancer 1 (H3K4me1+, H3K4me3+, H3K27ac+), Strong Enhancer 2 (H3K4me1+, H3K27ac+), Weak Enhancer (H3K4me1+), Transcription Transition (H3K36me3+, H3K4me1+, H3K27ac+/-), Transcription Elongation (H3K36me3+), Weak Transcription (H3K36me3+/-), H3K9me3 Repressed (H3K9me3+), H3K27me3 Repressed (H3K27me3+), Heterochromatin Low signal (none of the 6 marks). The p-value was calculated using the hypergeometric distribution which take into account the background of the array, when enrichment analysis was performed.

**Transcription Factor Binding sites and Pathway Enrichment Analysis**

The JASPAR 2018 database was applied for the detection of putative transcription factor binding sites on CpG sites using the R package TFBSTools . The function was applied on the human’s core collection of transcription factors, by setting the similarity threshold at least 80% with the methylated cytosine of each DMCpG expanded by 12bp on each side. The pathway enrichment analysis of the differentially methylated genes per case was performed using the R package “EnrichR” , based on the KEGG Pathway database. After the enrichment analysis, the p-values were calculated using the hypergeometric distribution which take into account the background of the array.

**Statistical analysis and visualization**

Data analysis was carried out in the R environment. The Pearson correlation coefficient (R) was used for the investigation of linear correlation. When linear relations were not observed, the Spearman coefficient (rho) was used instead. The standard chi-squared test was applied to evaluate the dependence between categorical variables (when not appropriate, the Fisher test was used instead). For both the time to first treatment (TTFT) and time to relapse (TTR), Kaplan-Meier curves were constructed to estimate the survival function.

**Supplementary Figures**


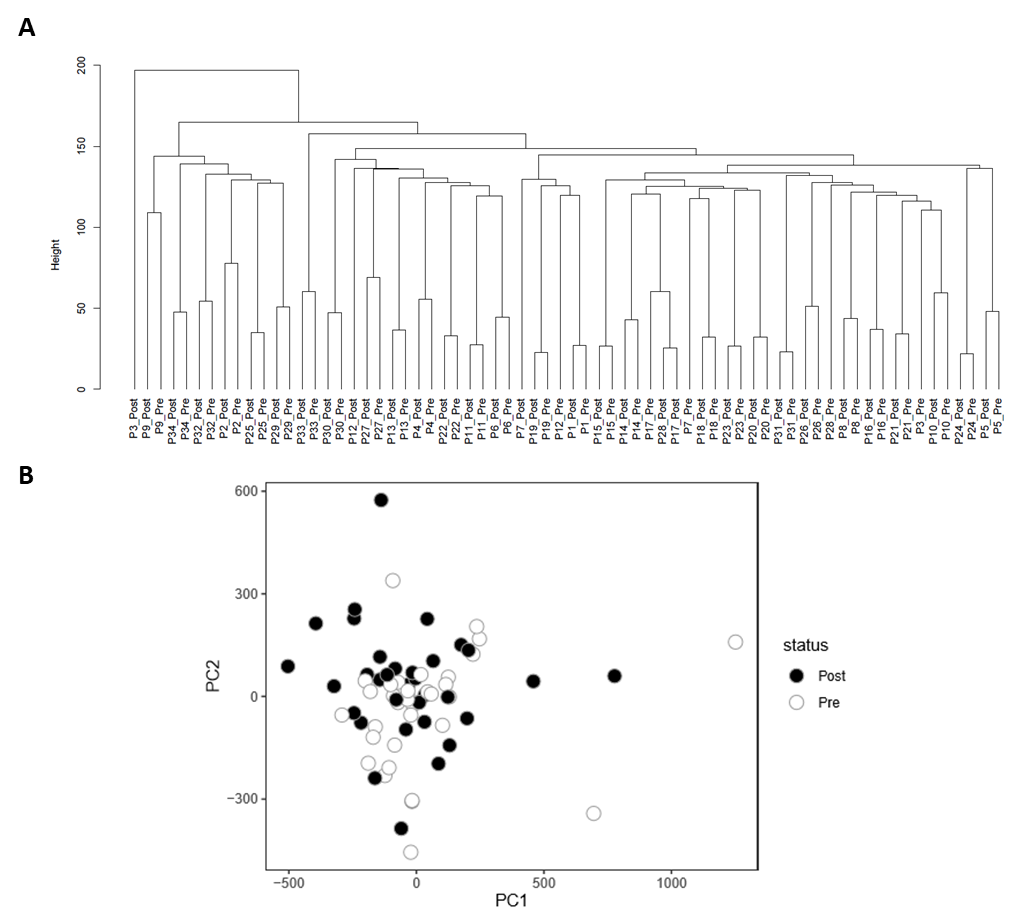


**Supplementary Figure 1: A.** Hierarchical clustering of all 68 samples, based on the methylation levels of 451756 CpG sites analyzed per case. 30/34 cases showed strong intra-individual similarity. **B.** Principal component analysis showing components 1 and 2 in the pre treatment and post relapse stage based on 451756 CpG sites.


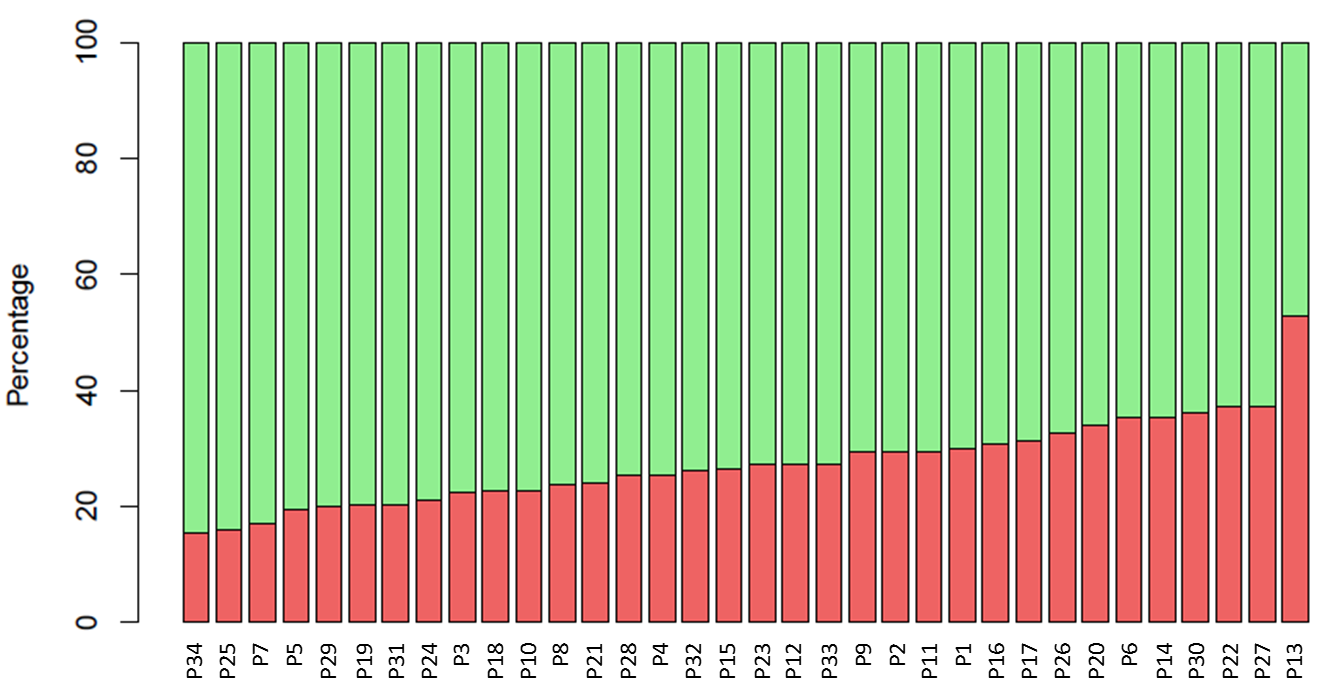


**Supplementary Figure 2**. Barplots showing the percentage of hypermethylated (red color) and hypomethylated (green color) CpG sites revealed after the comparison of the MBC (memory B cells) with the pre-treatment state of each case analyzed.

**
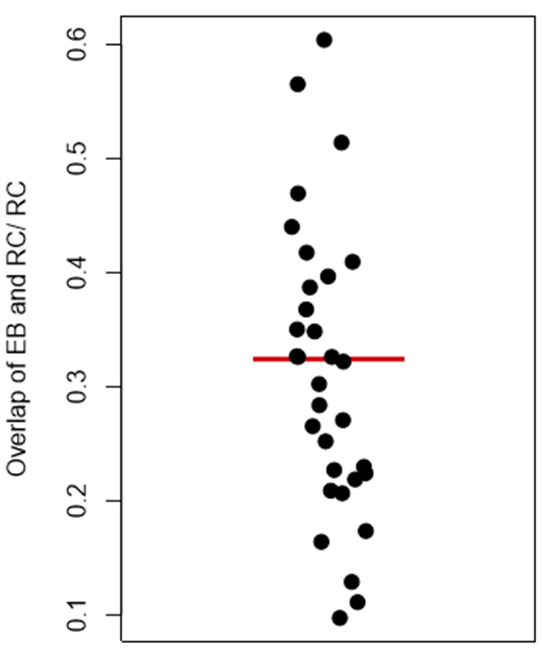
**

**Supplementary Figure 3.** Dot plots with median showing the RC in subgroups of CLL cases based on the genomic aberrations.

**
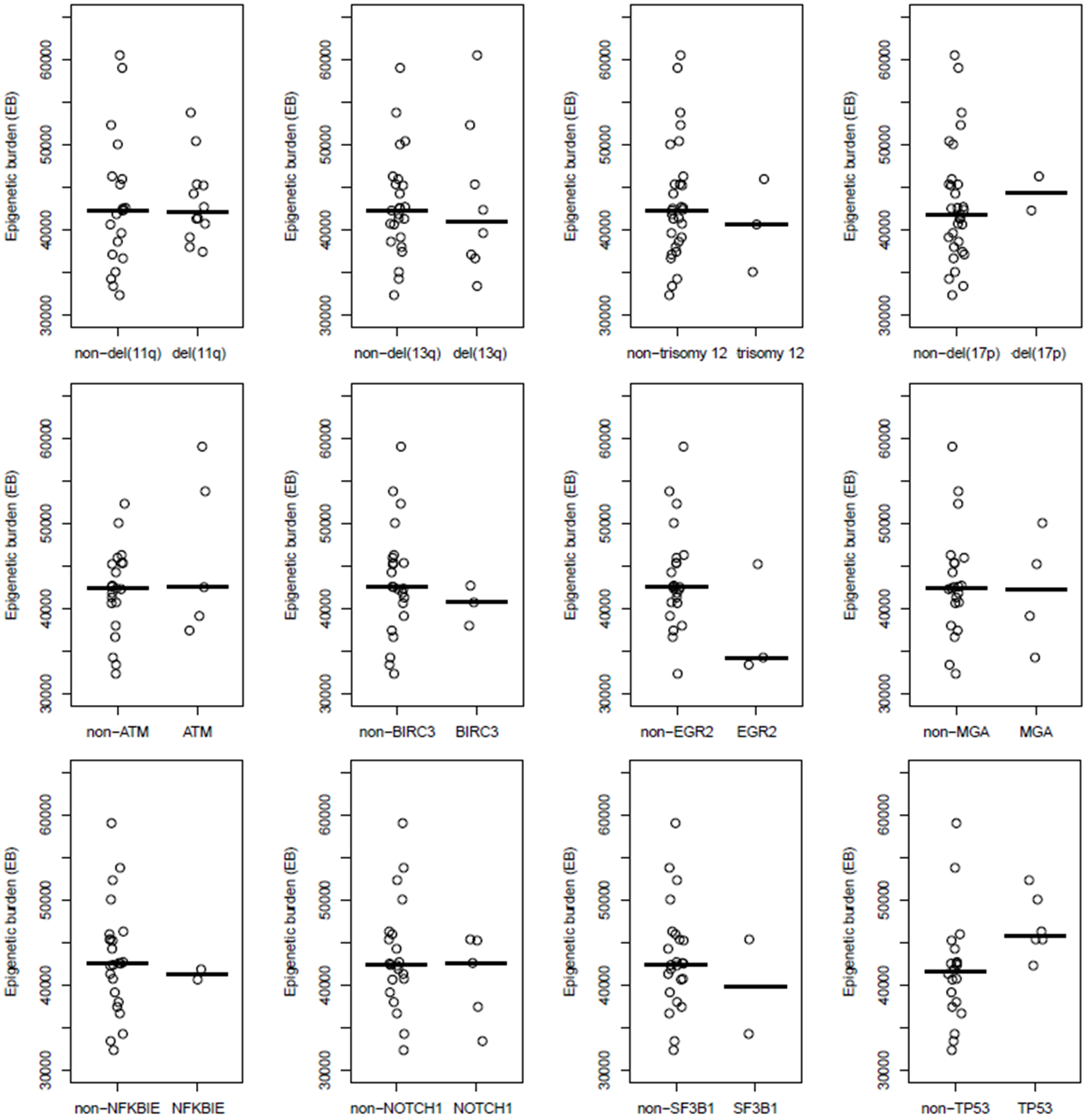
Supplementary Figure 4.** Dot plots with median showing the EB in subgroups of CLL cases based on the genomic aberrations.

**
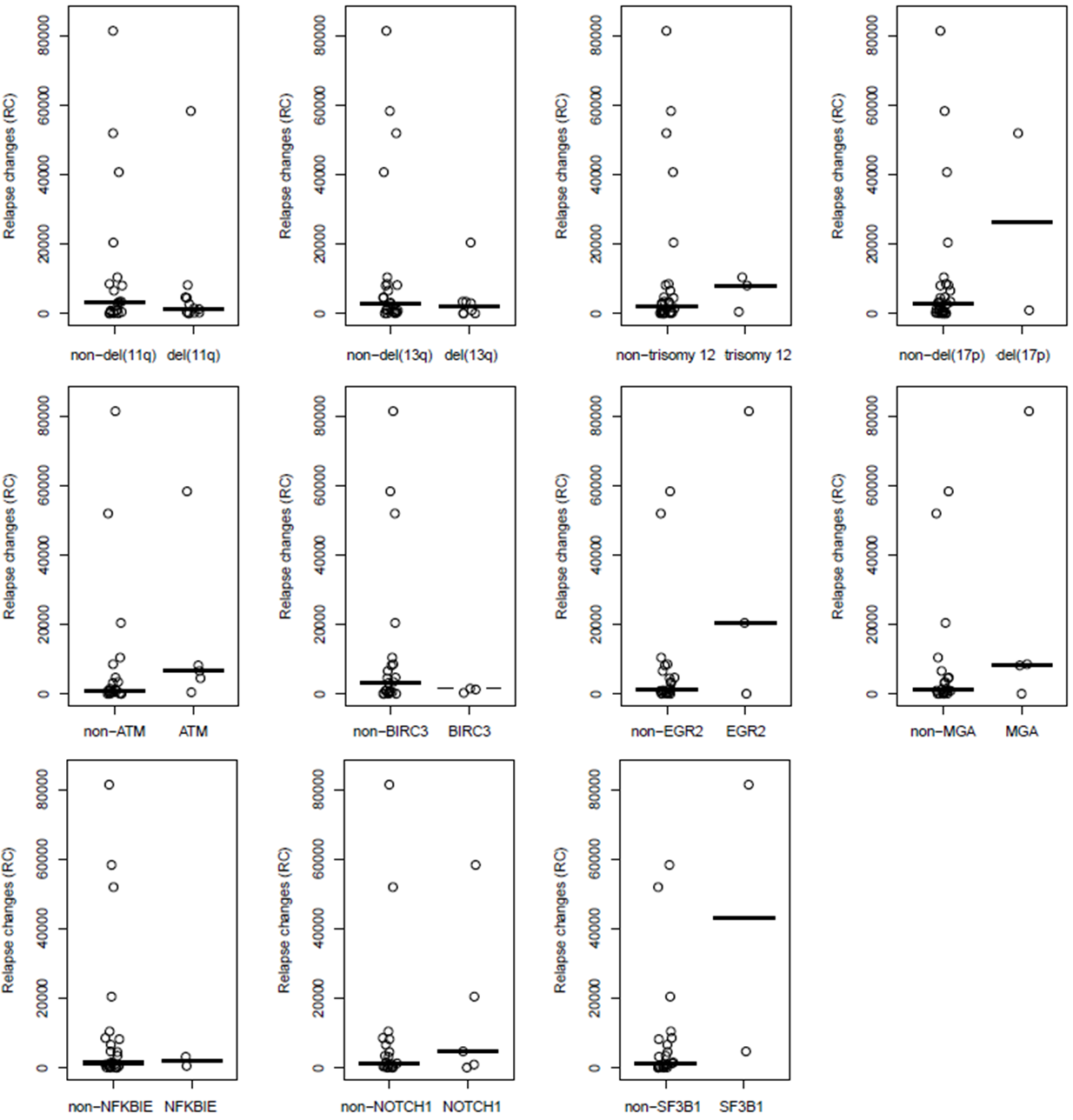
**

**Supplementary Figure 5.** Dot plots with median showing the RC in subgroups of CLL cases based on the genomic aberrations.


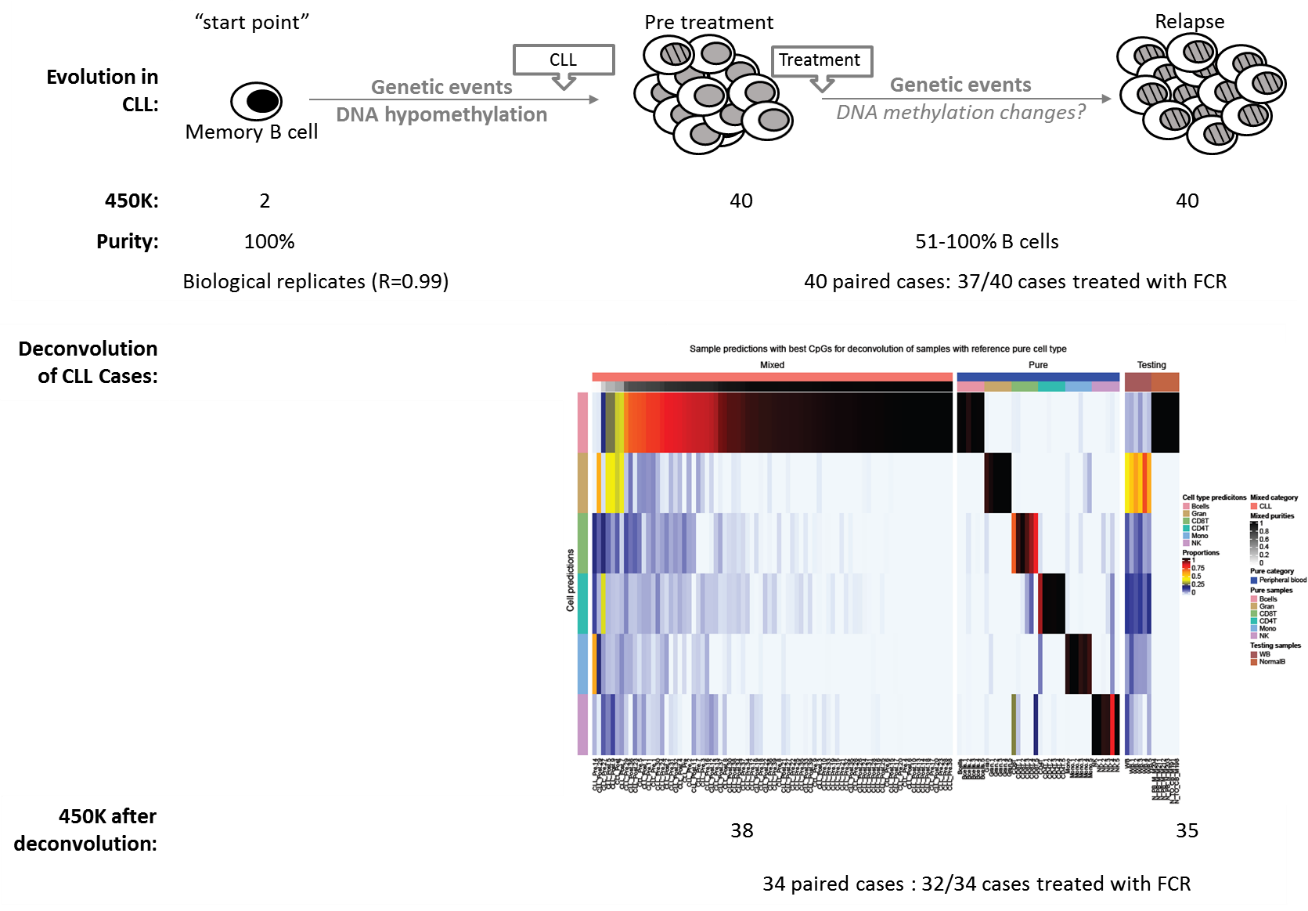


**Supplementary Figure 6**. Graphical description of the study aim, the study group and the methods used. We performed deconvolution of DNA methylation data, since a part of CLL samples was characterized bythe tumor load <95%. Estimation of the proportion of hematopoietic cell subpopulations in CLL samples and sorted B cells, CD8+ T cells, CD4+ T cells, natural killer cells, monocytes and granulocytes. Sorted cell subpopulations (a right part of the heatmap) are correctly predicted and CLL cases show a gradient from lower to higher proportion of B cells (a left part of the heatmap).

**References**

1. Queiros AC, Beekman R, Vilarrasa-Blasi R, Duran-Ferrer M, Clot G, Merkel A, et al. Decoding the DNA Methylome of Mantle Cell Lymphoma in the Light of the Entire B Cell Lineage. Cancer cell. 2016;30(5):806-21.

2. Ernst J, Kheradpour P, Mikkelsen TS, Shoresh N, Ward LD, Epstein CB, et al. Mapping and analysis of chromatin state dynamics in nine human cell types. Nature. 2011;473(7345):43-9.

3. Khan A, Fornes O, Stigliani A, Gheorghe M, Castro-Mondragon JA, van der Lee R, et al. JASPAR 2018: update of the open-access database of transcription factor binding profiles and its web framework. Nucleic acids research. 2018;46(D1):D260-D6.

4. Tan G, Lenhard B. TFBSTools: an R/bioconductor package for transcription factor binding site analysis. Bioinformatics. 2016;32(10):1555-6.

5. Chen EY, Tan CM, Kou Y, Duan Q, Wang Z, Meirelles GV, et al. Enrichr: interactive and collaborative HTML5 gene list enrichment analysis tool. BMC bioinformatics. 2013;14:128.
